# The Effect of Ambient Illumination and Text Color on Visual Fatigue under Negative Polarity

**DOI:** 10.3390/s24113516

**Published:** 2024-05-30

**Authors:** Qiangqiang Fan, Jinhan Xie, Zhaoyang Dong, Yang Wang

**Affiliations:** Department of Design, Northeastern University, Shenyang 110004, China; 2101335@stu.neu.edu.cn (J.X.); 20230851@stu.neu.edu.cn (Z.D.); 2171537@stu.neu.edu.cn (Y.W.)

**Keywords:** visual fatigue, ambient illumination, negative polarity, text color

## Abstract

This study investigates the effects of ambient illumination and negatively polarized text color on visual fatigue, exploring the issue of visual fatigue when using visual display terminals in low-illumination environments. The research methodology utilizes an experimental design to collect data on changes in pupil accommodation and blink rate through an eye tracker. Participants completed a reading task while exposed to various text colors and ambient light conditions to evaluate visual fatigue and cognitive performance. The study’s findings suggest that text color significantly affects visual fatigue, with red text causing the highest level of visual fatigue and yellow text causing the lowest level of visual fatigue. Improvements in ambient lighting reduce visual fatigue, but the degree of improvement varies depending on the text color. Additionally, cognitive performance is better when using yellow and white text but worse when using red text. Yellow text is the most effective choice for reducing visual fatigue under negative polarity. Increasing ambient lighting can also improve visual fatigue in low-illumination conditions. These findings will offer valuable guidance for designing visual terminal device interfaces, especially for low-illumination or night environments, to minimize visual fatigue and improve user experience.

## 1. Introduction

The effect of ambient illumination and text color on visual fatigue under negative polarity is a significant issue in the field of ergonomics, as the application of visual display terminals is becoming more widespread and more users are using them in different environments, which opens up a wider scope for research on visual fatigue. For example, with the development of electric vehicles, the combination of new technologies and the original hardware in the car driving space further promotes the smartness of the car and thus changes people’s driving habits. HUD is an essential product in this trend, which introduces reality enhancement technology to the original driving dashboard and HUD (Head-Up Display) and further improves the combination with the real environment based on the original instrumentation; the most important role of AR-HUD is to ensure the driver’s driving stability.The main function of the AR-HUD is to ensure the driver’s driving stability without having to look down at the dashboard and car navigation and to focus more on observing actual road conditions. However, at night, which is limited by the reduction in light intensity, drivers are prone to visual fatigue due to the overuse of vision when looking at the colorfully designed HUD interfaces, which affects driving performance and safety [1]. Thus, further research is necessary to explore the impact of color factors on visual fatigue under varying ambient illumination, particularly in complex human–computer interaction environments like HUDs. This research aims to provide specific theoretical and empirical support for the design of similar visual display terminal interfaces.

Visual fatigue is the most evident physiological manifestation of Computer Vision Syndrome, which includes ocular and systemic discomfort symptoms such as eye strain, dryness, blurred vision, and headaches resulting from prolonged use of computers or related electronic devices [2]. Particularly in low-ambient-illumination environments, the frequency of electronic device usage increases, exacerbating visual fatigue [3].

There is much research on the factors affecting visual fatigue, among which, to reduce visual fatigue at night (in low-illumination environments), more and more designers introduce negative polarity into the visual device terminals and use negative polarity as a new display color mode. Negative polarity, placing bright text on a dark background color, is also known as dark mode [4], while the opposite is positive polarity, with dark text on a bright background. 

Numerous studies have investigated factors affecting visual fatigue, with a growing interest in the effects of negative polarity display modes. The impact of negative polarity display modes on visual fatigue is increasingly attracting attention in ergonomics and visual health. Some studies have yielded conflicting results regarding the reduction in visual fatigue. Lin and Yeh’s research on TFT-LCD polarity and visual performance suggests that negative polarity may alleviate fatigue for specific visual tasks, particularly for older adults [5]. Erickson, Kim, Bruder, and Welch [3] investigated the relationship between color patterns, ambient illumination, visual acuity, and fatigue on virtual reality head-mounted displays (VR HMDs). Their findings indicate that negative polarity modes significantly reduce visual fatigue and enhance visual acuity in dimly lit virtual environments. Piepenbrock, Mayr, and Buchner [6] explored the relationship between display polarity and proofreading ability, revealing that participants performed better with positive polarity displays (light mode) than with negative polarity displays (dark mode). This suggests that negative polarity display modes can influence the user’s visual system and task performance. However, studies with differing conclusions argue that visual fatigue is multifaceted and related to various factors, including user preferences, and cannot be solely determined by display mode. Sethi and Ziat [7] used a combination of eye tracking and subjective data to show that dark mode (negative polarity displays) may increase the cognitive load for certain individuals, indicating a subtle interaction between user age, screen interface, and ambient illumination. Fan et al.’s [8] study on eye movement characteristics and visual fatigue in VR gaming, though not directly assessing dark mode, underscores the complexity of visual fatigue, hinting at influences such as interaction mode and gameplay duration. These findings suggest that the benefits of dark mode may depend on specific user characteristics and environments. A recent study [9] aimed to assess changes in pupil accommodation magnitude across different display polarities, finding that both positive and negative polarity conditions resulted in a significant decrease in pupil accommodation magnitude and an increase in visual fatigue symptoms. This research indicates that prolonged exposure to displays, regardless of polarity, may lead to visual stress. The aforementioned research highlights the multifaceted nature of negative polarity display modes’ impact on visual fatigue. While some studies demonstrate positive effects, the relationship between negative polarity display modes and visual fatigue is not straightforward, necessitating more comprehensive controlled studies to explore the effects of a broader range of variables on visual fatigue, including user preference, text type, ambient illumination, and the negative polarity modes themselves.

One of the most critical factors affecting visual fatigue beyond traditional display mode is ambient illumination, the general level of light in an environment provided by natural or artificial sources. It determines the overall brightness of a space and aids in perceiving surroundings. With the widespread adoption of negative polarity modes, particularly in electronic display terminals, some researchers find that increasing ambient illumination can mitigate visual fatigue. Lili Wang et al. [10] found that ambient illumination significantly affects visual fatigue while watching TV, with different levels leading to varying degrees of fatigue. They noted that watching TV in a dark environment typically results in more visual fatigue than in a light environment. Yang et al. [11] conducted an in-depth study on how ambient illumination and light flicker affect attention and visual fatigue in indoor environments, combining different illumination frequencies to observe their combined impact on users. Their results show that while illumination significantly affects attention and alertness, visual fatigue symptoms like ghosting and blurring are strongly correlated with light flicker frequency and ambient illumination intensity. This implies that both the intensity and quality of light can profoundly affect visual comfort and performance. Lin et al. [12] demonstrated that variations in color light and illumination levels significantly affect visual clarity and subjective visual fatigue, focusing on how VDT (Video Display Terminal) workstation illumination conditions affect the operator’s visual workload. They found that illumination level significantly affects changes in CFF (Critical Flicker Frequency) threshold and reaction time, providing initial insights into adjusting ambient illumination levels to reduce visual fatigue. Wang et al. [13] explored the effects of different ambient illumination sources and display types on visual fatigue through a complementary study, assessing visual fatigue through both subjective user ratings and objective ophthalmic parameters. They found that visual fatigue was particularly pronounced when watching 2D films in dark environments, highlighting the potentially detrimental effects of low ambient illumination on visual fatigue. These studies have focused on the impact of ambient illumination changes on visual fatigue in both bright and dark environments. However, more detailed research is needed on the effects of changing ambient illumination on visual fatigue in dark environments.

Regarding the display interface, color is an essential design element in electronic devices’ interactive interfaces and a necessary component in the field of traffic driving. Many studies have pointed out that color can significantly impact visual perception [14,15], yet most research on color’s effect on visual fatigue has focused on color temperature or illumination light color [16]. Wang, Chen, and Chen (2002) [17] investigated the impact of dynamic information display design on users’ visual performance and fatigue, finding that variables like speed, text/background color combinations, and jump length greatly affect subjects’ visual performance and fatigue levels. This study underscores the need for ergonomic design in digital displays to enhance readability and reduce visual discomfort. The researchers suggested that text and background colors are related to visual fatigue. Lin et al. further explored the role of colors in illumination and visual content engagement [18], discovering that yellow with red and blue with yellow result in more visual fatigue than combinations like yellow with black and white with blue. These insights are invaluable for designers and content creators, emphasizing the importance of selecting color schemes that minimize visual strain on viewers. In recent years, Li et al. [19] delved into the nuances of display choice, focusing on the impact of colored text against a black background on visual fatigue in display reading. Their findings support the idea that reading colored text on a black background provides a more comfortable visual experience than reading on a white background. Additionally, Lin and Feng evaluated visual workload under various VDT (Video Display Terminal) workstation lighting conditions, highlighting how color and illumination levels significantly affect visual sensitivity and subjective visual fatigue [12]. This study shows that there may be an interaction between color and ambient illumination.

The results of the aforementioned studies emphasize the multidimensional nature of visual ergonomics, suggesting that considering all these factors is vital for reducing visual fatigue and enhancing visual comfort in various environments. However, there is still space for further exploration of the topics covered in the studies. Firstly, most studies have yet to investigate the impact of ambient illumination and text color on visual fatigue, with research on the interaction between ambient illumination and color primarily focusing on indoor environments. Yet, visual display terminals such as HUDs, which are used in complex and changing environments, are constantly affected by the interaction of text color and ambient illumination. Secondly, most studies did not consider whether changes in ambient illumination in low-illumination environments affect visual fatigue under negative polarity. However, whether in an automotive driving space like a HUD or in an environment where visual display terminals are used at night, the actual habits of the user at night are often related to the changes in ambient illumination that the user experiences. Finally, although many studies have investigated visual fatigue by changing the ambient light level and text color, the results are mainly based on the user’s self-assessed visual fatigue index, and the objectivity of the results could be further enhanced.

Based on the existing literature, it is clear that there remains considerable scope for further inquiry into the effects of visual display parameters on ocular strain. This study is designed to probe into the following specific questions: How do text color and ambient light conditions influence visual fatigue under negative polarity settings, and what is the nature of the interaction between text color and ambient light? Given that users often operate in low-light conditions when employing negative polarity modes, this research prioritizes the examination of lower ambient light levels and the color palette commonly utilized in standard interface design as the primary variables. The screen luminance was meticulously regulated to 28 cd/m^2^ (representing 5% of maximum screen brightness, akin to the settings users typically prefer when engaging with electronic devices in dimly lit conditions), with the text background explicitly set to a uniform black. An eye tracker was deployed to monitor changes in pupil accommodation and blink rate, serving as indicators of visual fatigue, while the Likert Scale [20] was employed to gauge the subjective index of visual strain. By manipulating ambient light and color conditions, our research endeavors to quantify the variations in visual fatigue through both objective metrics and subjective assessments. The ultimate goal is to contribute to the development of more ergonomic interface designs for visual display terminals utilized in low-ambient-light settings and to establish a foundation for the creation of human–computer interaction devices in intricate environments, such as HUDs, that are designed to minimize visual fatigue and, by extension, the associated risks.

This dissertation posits the following hypotheses:

**H1.**  *Certain text colors are anticipated to alleviate visual fatigue in settings with diminished ambient light.*

**H2.**  *An increase in ambient light intensity is expected to correspond with a reduction in visual fatigue within low-light environments.*

**H3.**  *Specific text colors are predicted to exert an influence on cognitive performance under conditions of reduced ambient light.*

**H4.**  *Different ambient lighting conditions will interact with different text colors.*

The subsequent sections of this paper are structured as follows: Section 2 delineates the experimental methodology, Section 3 describes the analysis of the experimental data, Section 4 presents a discussion of the findings, and Section 5 provides a comprehensive conclusion to the study.

## 2. Materials and Methods

### 2.1. Experiment Design

The independent variables in this study are the text color and ambient illumination conditions under negative polarity. To investigate the changes in visual fatigue under negative polarity displays, we have identified text color and ambient illumination conditions as the independent variables. The experimental outcomes will be achieved by manipulating these two factors.

Ambient illumination intensity refers to the level of illumination in a given environment. Our experiments employed three distinct levels of ambient illumination intensity—0 lux, 5 lux, and 10 lux—to simulate nocturnal and twilight lighting conditions. Specifically, 0 lux represents the traditional illumination intensity of a completely dark environment, while 5 lux offers a slightly elevated level of ambient illumination. The 10 lux level, which is akin to indoor lighting at dusk, was informed by the research of Setyaningsih et al. [21]. Their study indicated that at an illumination intensity of 10 lux, the visibility of road infrastructure, mid-road barriers, potholes, and other vehicles is enhanced, making it the optimal ambient illumination intensity for nighttime driving. By establishing these three different illumination intensities, we aim to conveniently explore the variations in user visual fatigue at lower ambient illumination levels. Ambient illumination, in this context, is defined as the illuminance on the subject’s workbench. Two lamp fixtures are positioned above the workstation and meticulously adjusted to control the illumination according to the experimental conditions, complemented by a lampshade to prevent glare from affecting the displayed content. We hypothesize that different ambient illumination conditions will exert a reciprocal influence on text color.

**Figure 1:**
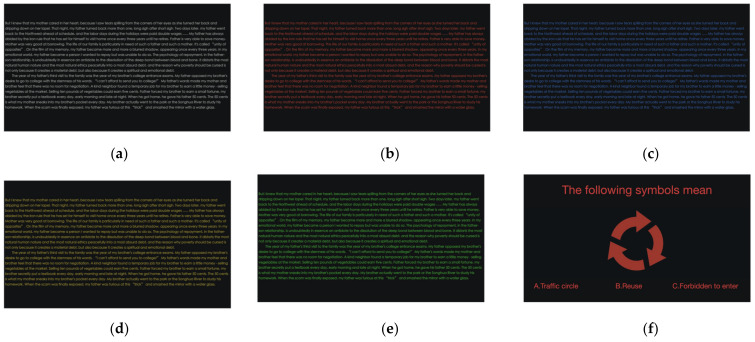
The different experimental conditions of text and symbols. Five kinds of text colors are shown in (**a**–**e**); the styles of the symbols are shown in (**f**).

In terms of text color variable design, colors can be defined in various ways, including RGB and HBS models. Our study adopts the HBS value as the primary measure, as this color model is standard for translating colors in computer devices. We utilize the hue value of the text color as the variable, while the brightness (B) and saturation (S) of the text color are held constant. Initially, we determined the background color; for OLED screens, dark mode is set to pure black [22] (no light emitted in the background); thus, we selected pure black (H: 0% S: 0% B: 0%). Subsequently, in accordance with WCAG [23], which stipulates that the luminance contrast of visual display terminals should be at least 1:3, we chose four colors frequently used in interface design and favored by users [24]: blue (H: 218° S: 70% B: 62%), red (H: 3° S: 70% B: 62%), yellow (H: 49° S: 70% B: 62%), and green (H: 113° S: 70% B: 62%). The brightness and saturation of these colors are uniform. For comparative analysis with other color combinations, an additional control option was introduced, selecting white (H: 0° S: 0% B: 95%) with a luminance contrast (Michelson contrast) of 0.969 against the background, which is deemed a less visually fatiguing choice [25]. The HSB and RGB values of the colors and their luminance contrasted with the background color are presented in Table 1. In the experiment, the five independent color variables are represented as text and specific symbols, which will be detailed in the subsequent experimental procedure. The various experimental conditions for text and symbols are depicted in Figure 1.

In this study, we devised a comprehensive experimental matrix consisting of 15 (5 × 3 treatments) unique conditions that combined different text colors with varying levels of ambient illumination. The participant pool, comprising 50 individuals, was evenly distributed across five groups, with a balanced representation of both genders. Each group was assigned to one of the five text color categories, which served as the between-subjects variable. Over the course of three consecutive days, each participant underwent a series of ambient illumination treatments, with each day presenting a different illumination level: 0 lux, 5 lux, and 10 lux. This sequence of ambient light conditions represented the within-subject variable, ensuring that each participant experienced all three illumination levels in a counterbalanced manner. This rigorous experimental design allows for a thorough examination of the interactive effects between text color and ambient light on visual fatigue.

### 2.2. Subjects 

In our study, we enlisted a cohort of 50 participants, comprising 27 males and 23 females. The majority were affiliated with Northeastern University, while the remaining subjects were online car drivers. The age distribution of our participants spanned from 20 to 32 years old (M = 27, SD = 4). An experienced tester utilized the Tumbling E chart [26] to assess the visual acuity of each subject. All participants possessed normal or corrected-to-normal vision, with a visual acuity of 1.0 or better (in decimal units), and they all passed a stringent test for color blindness to ensure their sensitivity to color was adequate. Each participant had the necessary visual, reading, and cognitive skills acquired through primary compulsory education and had successfully obtained a driver’s license.

Prior to the commencement of the experiment, participants were provided with a comprehensive briefing by the experimental trainer, followed by a rigorous training session to familiarize them with the procedures. Subsequently, the subjects engaged in a rigorous simulation. They were instructed to ensure adequate rest and sleep to minimize the impact of extraneous variables, such as sleep deprivation, on the experimental outcomes.

### 2.3. Apparatus

For our visual display apparatus, we employed a Lenovo M14D monitor with a 16:9 aspect ratio, a resolution of 2240 × 1400, and a refresh rate of 75 Hz. The eye-tracking component was facilitated by a Tobii Pro X3-120 (Danderyd Municipality, Sweden), which boasts a 16:9 aspect ratio, a resolution of 2240 × 1400, and a refresh rate of 75 Hz. The eye tracker operates at a sampling frequency of 120 Hz, with a sampling accuracy of 0.3 degrees, and allows for a head movement range of 10 cm × 50 cm × 40 cm. The convenience of the subjects was prioritized by placing the eye tracker directly beneath the screen, eliminating the need for it to be worn.

We regulated the ambient illumination in the experimental space using an intelligent LED lamp set to a color temperature of approximately 5100 K. We used the intelligent LED lamp MJTD06YL (Xiaomi Corporation, Beijing, China); the spectrum of the lamp is RA = 97, which is close to a full-spectrum LED light. The ambient illuminance was measured at the position of the eyes with the display off by a TASI TA 630A/B (TASI Corporation, Suzhou, China) handheld digital illuminance meter.

### 2.4. Conditions of the Workplace

**Figure 2:**
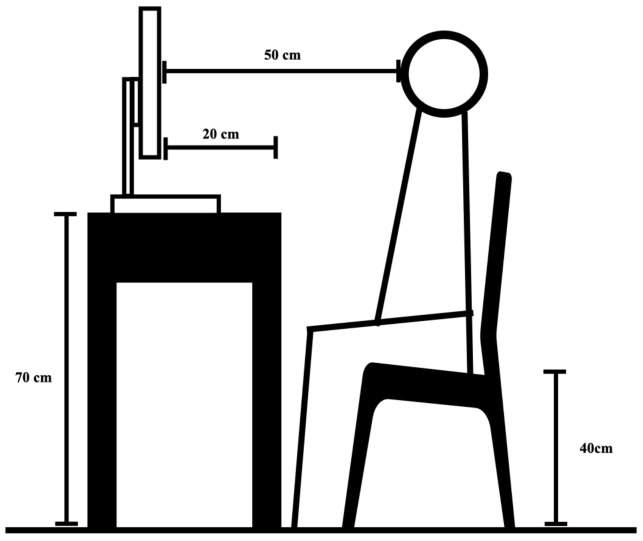
Experimental environment and specific parameters of laboratory.

As shown in the picture (Figure 2), in order to simulate a completely dark experimental environment, we utilized a dedicated laboratory equipped with a specialized experimental setup. The workspace was furnished with a table at a height of 73 cm, a monitor equipped with a sunshade, and a chair adjustable to a height of 42 cm. The monitor’s height is adjustable between 40 cm and 50 cm. The monitor was positioned 20 cm from the edge of the table to the screen’s center, with a 90-degree tilt perpendicular to the table surface. The monitor’s height was customized according to each subject’s height, following the user guidelines of the Tobii eye-tracking device. The horizontal distance from the center of the screen to the subject’s eyes was maintained at approximately 55 cm. The intelligent LED lamp was placed at the top of the lab, above the subjects’ seats. The intelligent LED lamp was kept at a distance of nearly two meters from the subjects.

### 2.5. Task and Procedure

**Figure 3:**
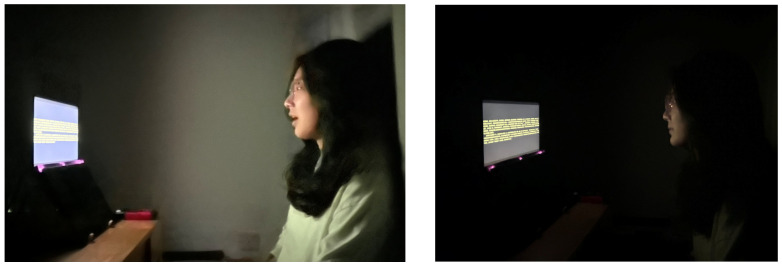
Experimental procedures under different conditions.

Participants in our study were required to complete an online consent form, and those deemed suitable were invited to contribute to our experiment. Upon their arrival, subjects were gently guided through the process by our team of professional experiment trainers, who provided a thorough overview of the entire experimental procedure. In a preparatory room, our trainers facilitated a brief pre-experiment exercise for the subjects, which involved text reading and symbol recognition tasks. This initial activity served to familiarize participants with the nature of the tasks they would be performing. Subsequently, subjects were escorted into a darkened environment designed to mimic the low-illumination conditions of the actual experiment. This allowed them to acclimate in advance, ensuring comfort and readiness for the experimental conditions. Here, they were invited to take a seat in front of the display and make their eyes relaxed, where they were guided to adopt an appropriate sitting posture and to establish the optimal viewing distance from the screen. Throughout this preparatory phase, our experimental assistants were on hand to offer assistance and ensure that each subject was well prepared for the main experiment. With all preparations in place, we then proceeded to the formal experimental phase. Figure 3 provides a visual representation of the actual experimental procedure, illustrating the steps taken to ensure a smooth and efficient experience for our participants.

Initially, participants were guided through a 5-point calibration test to ensure the accuracy of the eye-tracking equipment. Following this calibration, they were introduced to the primary task at hand—a reading assignment. The goal of our experiment was to gather ocular data while subjects engaged in reading, and to this end, we asked them to read aloud, ensuring a high level of focus and engagement throughout the task [27,28]. The reading material was an 8104-word Chinese novel selected for its neutral content, primarily consisting of the author’s personal family anecdotes. This choice was made to minimize any potential influence of the text’s emotional tone on the visual data collected. The novel was formatted into a 20-page text, with approximately 400 words per page, designed to be read in about 20 min at a typical reading pace. Given that negative polarity is more sensitive to text size than positive polarity [29] and recognizing that legibility can vary with different fonts, we selected a standard, bold font at 20 points, with 1.5 times the usual line spacing. This formatting was chosen to correspond with the subject’s behavioral parameters during testing, aiming to achieve an optimal visual angle of 20.5 min of arc for the bold font [30]. Upon completing the reading task, participants moved on to Task 2, which involved a cognitive test. In this task, a series of questions, traffic signs, or interface design symbols, colored to match the text from Task 1, were presented on the screen (Figure 1f). Participants were instructed to use the left mouse button to select the option they believed to be correct. This process was repeated for a total of 50 questions, and the accuracy of their responses was recorded to assess cognitive performance.

**Figure 4:**
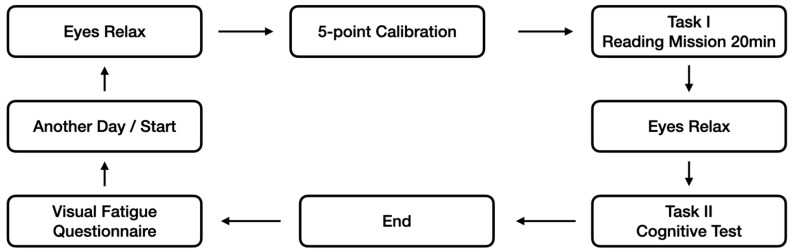
Experimental process.

After all visual tasks were completed, subjects were asked to complete a questionnaire to gauge their level of visual fatigue. For the second and third days of the experiment, ambient illumination conditions were modified, while the procedures followed on these days replicated those of the initial day. Upon the conclusion of the study, each participant received a stipend of RMB 50. The reading task required approximately 20 min each day, and the entire experimental process, including calibration and cognitive testing, took about 40 min daily. The experiment spanned three days, and the complete flowchart is depicted in Figure 4.

### 2.6. Dependent Variables

In our experiment, we chose a similar research approach to Xie et al. [25], in which objective and subjective indicators were used together as dependent variables to test the degree of visual fatigue from the subjects’ ocular physiological performance and subjective feelings. Objective indicators include pupil accommodation and blink rate, and subjective indicators include cognitive performance and visual fatigue index.

#### 2.6.1. Pupil Accommodation

Pupil accommodation is the rate of change of the pupil within a certain period and the ability of the pupil to adjust itself. A smaller pupil accommodation and a lower rate of change mean a slower adjustment of the pupil size and a decrease in its ability to regulate, which indicates an increase in the degree of visual fatigue and vice versa [31]. We used linear regression to calculate pupil diameter fluctuation over time for pupil accommodation. The size of the pupil diameter after the start of the experiment was regressed by linear regression, and the resulting regression coefficient is the pupil change rate, which represents the pupil’s ability to regulate.

#### 2.6.2. Blink Rate

Blink rate, characterized by the rapid closing and opening of the eyelids, is a critical eye movement that has been extensively studied in the context of visual fatigue. The spontaneous blink rate (SBR), which is the frequency of blinks over a set period, is a recognized measure of visual strain [32]. It is well-documented that prolonged use of electronic visual display terminals in dimly lit environments can lead to a decreased SBR [33]. This reduction in blinking is associated with increased corneal exposure time, potentially resulting in dry eyes—a significant contributor to visual fatigue. Consequently, a lower blink rate over an extended period of display usage is indicative of heightened visual fatigue. In our study, we quantified the blink rate by calculating the ratio of the total number of blinks recorded after the experiment started to the total duration of the experiment in minutes. 

#### 2.6.3. Cognitive Performance

Cognitive performance encompasses a wide range of mental processes, including memory, attention, executive function, and the ability to apply logical reasoning and problem solving [34]. On one hand, visual fatigue’s effects on cognitive performance are multifaceted and not limited to cognitive impairment. Excessive visual stimulation and prolonged periods of concentration can lead to changes in brain activity that can affect an individual’s memory, attention, and other cognitive functions [35,36]. Therefore, as visual fatigue increases, there is a significant decrease in an individual’s cognitive ability. This study defined our cognitive performance as the correct rate of quickly recognizing traffic and interface design symbols. In Task 2, there were 50 symbol recognition questions, and each correct one was awarded one point, and no points were counted for errors; all correct ones were awarded a total of 50 points, and all incorrect ones were awarded a total of 0 points. In the symbol recognition process, the statistics of using the mouse to click on the options after the symbols have been displayed for 3 s are regarded as invalid data, and the invalid data are also not counted in the total score. The lower the score, the higher the visual fatigue, and vice versa.

#### 2.6.4. Visual Fatigue Index

The visual fatigue index was derived from the questionnaire developed by Heuer and Hollendiek [37] and adapted with a 7-point Likert scale to suit our study. The questionnaire, consisting of 10 questions about visual perception and mental state, used a response scale ranging from “1.completely agree” to “7.completely disagree”. In this study, each question was scored as an option, with one indicating that the subject chose “completely agree” for one point and seven indicating they chose “completely disagree” for seven points. The average score from these questions is determined by the final visual fatigue index, with a lower index reflecting higher levels of visual fatigue. After the completion of all visual tasks, participants were asked to complete the visual fatigue questionnaire to ensure the reliability of our findings.

## 3. Results

We enlisted a team of 50 experimenters to conduct a total of 150 experimental trials. The validity of the ocular data collected was found to be 82.4%, which was not significantly different from the 83.1% validity rate observed during the five-point calibration phase. For our subjective, dependent variables, we ensured the reliability of the questionnaire to guarantee accurate survey responses. In our analysis, we initially employed a two-way multivariate analysis of variance (MANOVA) to assess the interaction effects between the two independent variables. The significance level was set at 0.05, indicating that an effect is considered significant if *p* < 0.05. For each independent variable, we further utilized Duncan multiple paired comparisons and Bonferroni multiple comparisons to explore the sources of significance and the correlations among the dependent variables. In cases where a significant interaction between two variables was detected, a simple effects analysis was conducted.

The results of our data analysis, covering pupil accommodation, blink rate, cognitive performance, and visual fatigue index, are detailed in the subsequent sections. Table 2 provides the means and standard deviations of the dependent variables across different levels of the independent variables.

### 3.1. Pupil Accommodation

The MANOVA results indicated that both text color [F (4, 145) = 360.687, *p* < 0.05, η^2^ = 0.914] and ambient illumination [F (2, 147) = 17.082, *p* < 0.05, η^2^ = 0.202] had a significant impact on pupil accommodation, with no significant interactions observed between different text colors and ambient illumination changes [F (8, 141) = 1.824, *p* > 0.05, η^2^ = 0.98].

**Figure 5:**
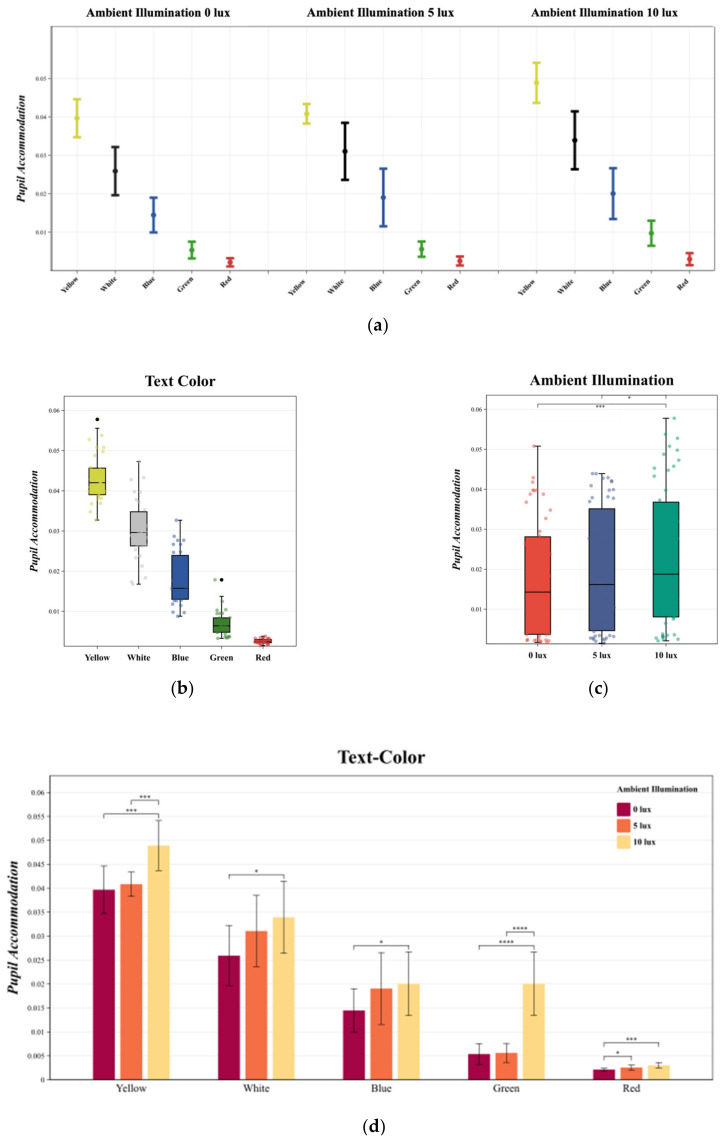
(**a**) The mean and standard deviation of pupil accommodation is described. Statistics were performed using MANOVA. (**b**) describes the changes in pupil accommodation under different text colors. Duncan multiple paired comparisons were used for the statistics. (**c**) describes the changes in pupil accommodation under different ambient illuminations. Bonferroni multiple comparisons were used for the statistics. (**d**) describes the interaction between text color and ambient illumination in pupil accommodation changes. Bonferroni multiple comparisons were used for the statistics. **** *p* < 0.0001; *** *p* < 0.001; * *p* < 0.05.

The variations in pupil accommodation under different experimental conditions are depicted in Figure 5a.

Duncan multiple paired comparisons revealed that, for text color, the yellow text exhibited the highest pupil accommodation in the negative polarity display mode, irrespective of ambient illumination changes, followed by gray, blue, green, and red (Figure 5b). All five text colors demonstrated significant differences in pupil accommodation (*p* < 0.05). Regarding ambient illumination, the lowest pupil accommodation was observed at 0 lux, with a significant increase at both 5 lux and 10 lux as the ambient illumination intensified. Bonferroni multiple comparisons for ambient illumination and pupil accommodation showed that the difference between 0 lux and 10 lux was statistically significant (*p* = 0.001 < 0.05), while the difference between 0 lux and 5 lux was not significant (*p* = 0.055 > 0.05). The difference between 5 lux and 10 lux was also statistically significant (*p* = 0.02 < 0.05) (Figure 5c).

In examining the interaction between text color and ambient illumination, Bonferroni multiple comparisons revealed significant relationships as illustrated in Figure 5d. For the yellow text, an increase in ambient illumination from 5 lux to 10 lux resulted in a significant change in pupil accommodation. Conversely, the change from 0 lux to 5 lux did not yield a significant effect. Similarly, for the green text, a significant change in pupil accommodation was observed with an increase from 5 lux to 10 lux. In the case of the blue text, no significant change was noted when ambient illumination increased from 5 lux to 10 lux, but a change from 0 lux to 5 lux did result in a significant pupil accommodation response. For the white text, a consistent increase in pupil accommodation was noted with an increase in ambient illumination from 0 to 10 lux, indicating a correlation. Overall, there was a positive correlation between ambient illumination and pupil accommodation. For the red text, a non-significant change in pupil accommodation occurred with an increase from 0 lux to 5 lux. However, a significant change was observed with an increase from 0 lux to 10 lux. In summary, with 10 lux of ambient illumination, the yellow text resulted in the highest pupil accommodation, while the red text exhibited the lowest, regardless of the ambient illumination level.

### 3.2. Blink Rate

**Figure 6:**
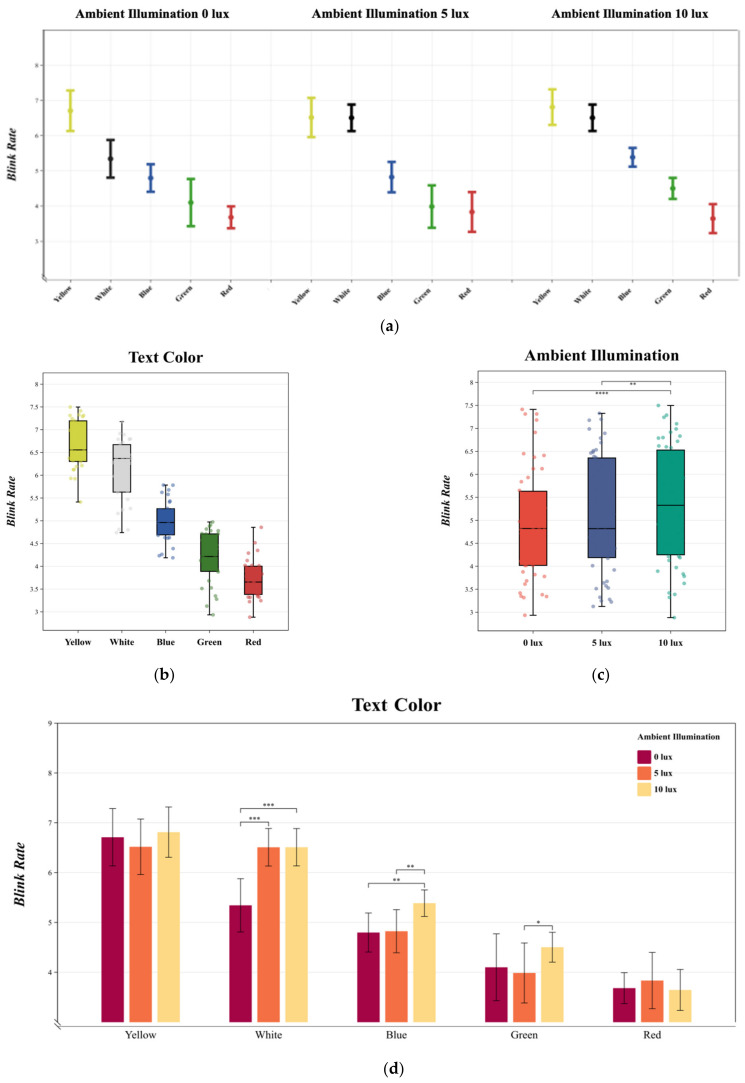
(**a**) The mean and standard deviation of blink rate in subjects is described. Statistics were performed using MANOVA. (**b**) describes the changes in blink rate under different text colors. Duncan multiple paired comparisons were used for the statistics. (**c**) describes the changes in blink rate under different ambient illuminations. Bonferroni multiple comparisons were used for the statistics. (**d**) describes the interaction between text color and ambient illumination in blink rate changes. Bonferroni multiple comparisons were used for the statistics. **** *p* < 0.0001; *** *p* < 0.001; ** *p* < 0.01; * *p* < 0.05.

The MANOVA results indicated that both text color [F (4, 145) = 209.201, *p* < 0.05, η^2^ = 0.861] and ambient illumination [F (2, 147) = 11.043, *p* < 0.05, η^2^ = 0.141] significantly influenced the blink rate. Additionally, a significant interaction was observed between text color and ambient illumination [F (8, 141) = 4.705, *p* < 0.05, η^2^ = 0.218]. The variations in blink rate under different experimental conditions are depicted in Figure 6a.

Duncan multiple paired comparisons revealed that for text color, in the negative polarity display mode, yellow text elicited the highest blink rate, followed by gray, blue, green, and red, irrespective of changes in ambient illumination (Figure 6b). Regarding ambient illumination, the lowest blink rate was observed at 0 lux, with a significant increase at both 5 lux and 10 lux as ambient illumination intensified. Bonferroni multiple comparisons for ambient illumination and blink rate showed that the difference between 0 lux and 10 lux was statistically significant (*p* = 0.001 < 0.05), while the difference between 0 lux and 5 lux was not significant (*p* = 0.090 > 0.05). The difference between 5 lux and 10 lux was also statistically significant (*p* = 0.04 < 0.05) (Figure 6c).

In examining the interaction between text color and ambient illumination, according to Bonferroni multiple comparisons (Figure 6d), no significant change in blink rate was noted for yellow text when ambient illumination increased from 0 lux to 10 lux. A similar trend was observed for red text, where the change from 0 lux to 10 lux did not significantly affect the blink rate. For white text, an increase in ambient illumination from 0 lux to 5 lux led to a significant rise in blink rate, a pattern also seen with red text, which showed a significant change in blink rate when ambient illumination increased from 0 lux to 10 lux, although the change from 5 lux to 10 lux was not significant. In the case of blue text, an increase in ambient illumination from 5 lux to 10 lux induced a significant change in blink rate, whereas the change from 0 lux to 5 lux was not significant. For green text, an increase from 5 lux to 10 lux caused some variation in blink rate, but the change from 0 lux to 5 lux did not result in a significant increase and even led to a decrease. Overall, yellow and white text at 5 lux and 10 lux across all ambient illumination conditions resulted in the highest blink rates, while red text consistently had the lowest blink rate, being particularly low at 10 lux.

### 3.3. Cognitive Performance

**Figure 7:**
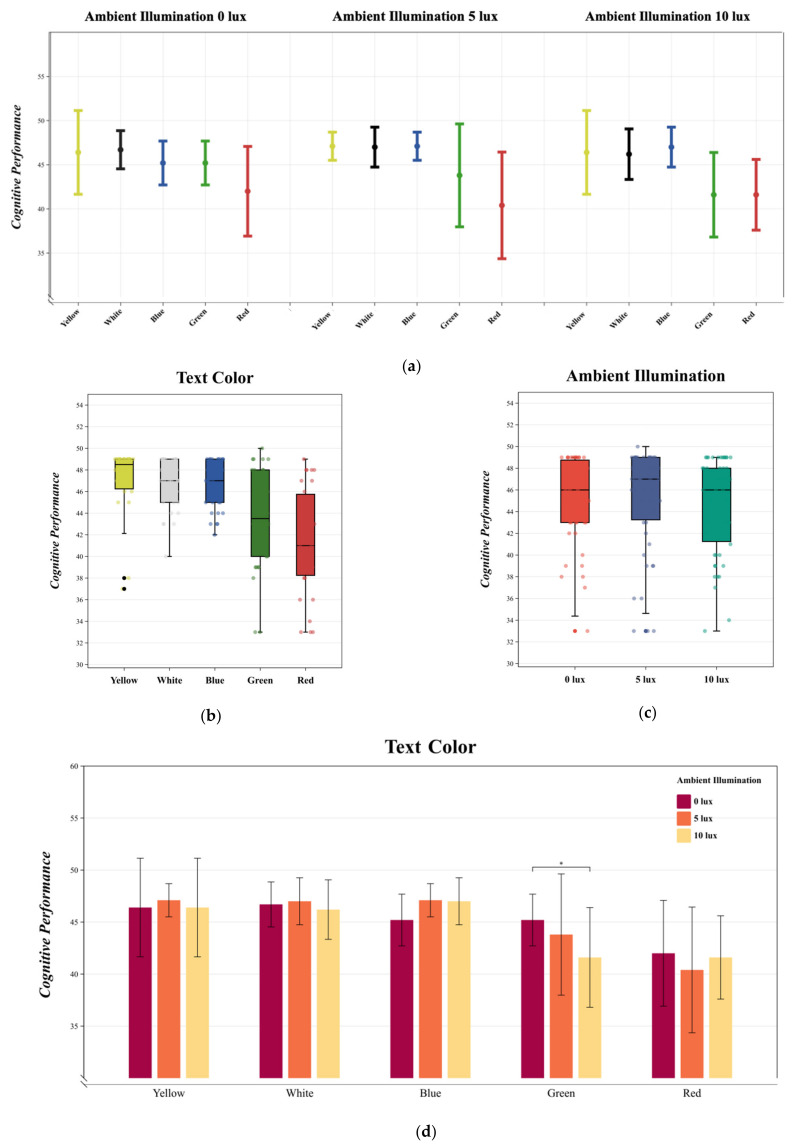
(**a**) The mean and standard deviation of cognitive performance is described. Statistics were performed using MANOVA. (**b**) describes the changes in cognitive performance under different text colors. Duncan multiple paired comparisons were used for the statistics. (**c**) describes the changes in cognitive performance under different ambient illuminations. Bonferroni multiple comparisons were used for the statistics. (**d**) describes the interaction between text color and ambient illumination in cognitive performance changes. Bonferroni multiple comparisons were used for the statistics. * *p* < 0.05.

MANOVA results indicated that text color [F (4, 145) = 117.999, *p* < 0.05, η^2^ = 0.257] significantly influenced cognitive performance, while ambient illumination [F (2, 147) = 0.319, *p* > 0.05, η^2^ = 0.005] did not. Additionally, no significant interactions were observed between text color and ambient illumination [F (8, 141) = 0.848, *p* > 0.05, η^2^ = 0.048]. The variations in the cognitive performance under different experimental conditions are depicted in Figure 7a.

Duncan multiple paired comparisons revealed that, for text color, red and green text stood out with significant differences in cognitive performance compared to the other three color groups in the negative polarity display mode. In contrast, yellow, blue, and white text did not exhibit significant differences in cognitive performance (Figure 7b). Regarding ambient illumination, cognitive performance was highest at 5 lux. There was no significant difference in mean cognitive performance scores between 0 lux and 10 lux ambient illumination, although the scores showed more variation under 10 lux conditions. The Bonferroni multiple comparisons between ambient illumination and cognitive performance did not yield significant statistical differences between 0 lux, 5 lux, and 10 lux, suggesting that ambient illumination did not have a significant impact on cognitive performance (Figure 7c).

In the interaction between text color and ambient illumination, as per Bonferroni multiple comparisons, no significant differences in cognitive performance were noted for yellow, white, and blue text across different levels of ambient illumination, which did not exhibit a consistent pattern of change (Figure 7d). For green text, an increase in ambient illumination from 0 lux to 10 lux resulted in a change in cognitive performance; although not significant, there was a negative correlation between ambient illumination and cognitive performance. For red text, a decrease in ambient illumination from 0 lux to 5 lux was associated with a negative correlation with cognitive performance, but an increase from 0 lux to 10 lux led to a significant change in cognitive performance. Overall, cognitive performance for yellow, blue, and white text did not significantly differ and was higher compared to other colors, with yellow text showing the best cognitive performance at 5 lux ambient illumination. Yellow text exhibited the highest cognitive performance at 5 lux, while red text performed the poorest at the same ambient illumination level.

### 3.4. Visual Fatigue Index

**Figure 8:**
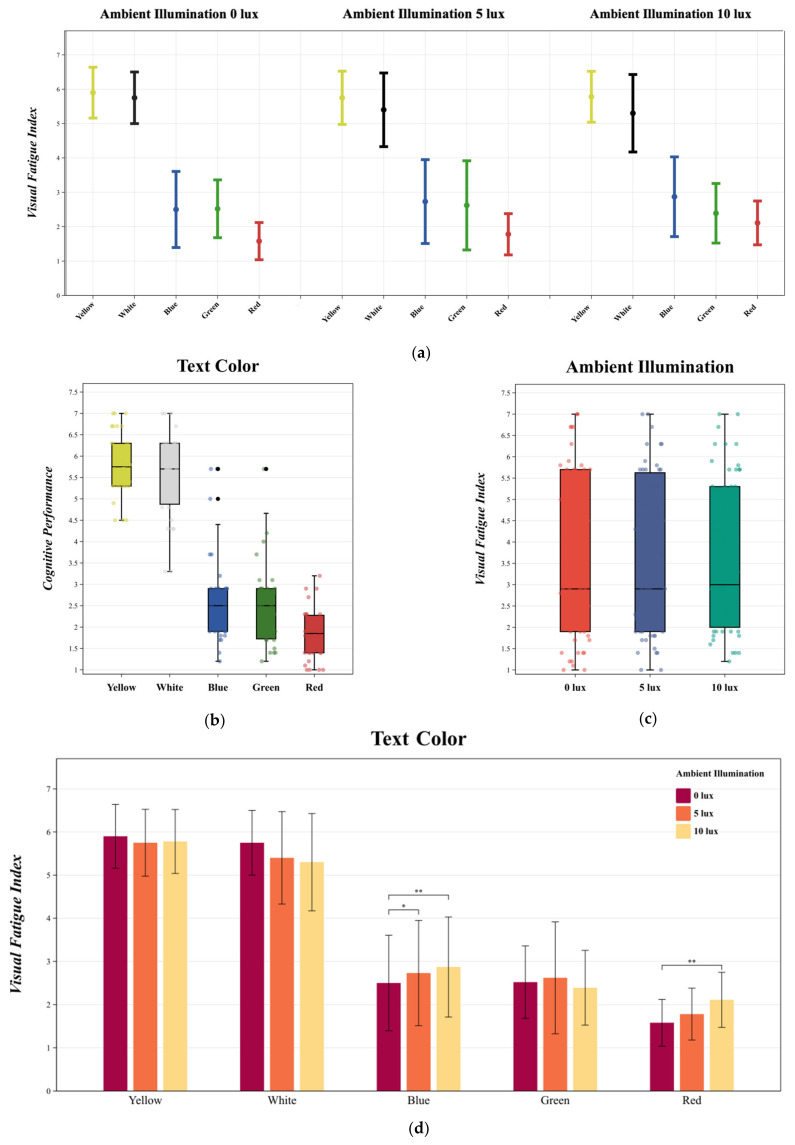
(**a**) The mean and standard deviation of the visual fatigue index is described. Statistics were performed using MANOVA. (**b**) describes the changes in visual fatigue index under different text colors. Duncan multiple paired comparisons were used for the statistics. (**c**) describes the changes in visual fatigue index under different ambient illuminations. Bonferroni multiple comparisons were used for the statistics. (**d**) describes the interaction between text color and ambient illumination in visual fatigue index changes. Bonferroni multiple comparisons were used for the statistics. ** *p* < 0.01; * *p* < 0.05.

MANOVA results indicated that changes in text color [F (4, 145) = 117.999, *p* < 0.05, η^2^ = 0.778] significantly affected the visual fatigue index, while ambient illumination [F (2, 147) = 0.027, *p* > 0.05, η^2^ = 0.00] did not. No significant interaction was found between text color and ambient illumination [F (8, 141) = 0.521, *p* > 0.05, η^2^ = 0.030]. The variations in the visual fatigue index under different experimental conditions are depicted in Figure 8a.

Duncan multiple paired comparisons revealed that, concerning text color, yellow text had the highest visual fatigue index in the negative polarity display mode, followed by white, blue, green, and red text (Figure 8b). There was no significant difference in the visual fatigue index between yellow and white text, and similarly, no difference was found between blue and green text. Regarding ambient illumination, the three levels tested did not show significant differences in the visual fatigue index. The Bonferroni multiple comparisons between ambient illumination —0 lux, 5 lux, and 10 lux—and the visual fatigue index indicated that ambient illumination did not yield significant statistical differences in the visual fatigue index (Figure 8c).

In the interaction between text color and ambient illumination, as per Bonferroni multiple comparisons, the visual fatigue index for yellow, white, and green text did not significantly change with ambient illumination (Figure 8d). However, for blue text, there was a positive correlation between ambient illumination and the visual fatigue index, meaning that the index increased significantly as ambient illumination rose from 0 lux to 10 lux. A similar trend was observed for red text, where the visual fatigue index increased significantly with an increase in ambient illumination from 0 lux to 10 lux. Overall, the visual fatigue indices of yellow and white text were not significantly different and were higher compared to other colors, with yellow text exhibiting the highest visual fatigue index at 0 lux ambient illumination. There was no significant difference between blue and green text, while red text had the lowest visual fatigue index at 0 lux ambient illumination.

Pearson correlation analysis was used to test the correlation between the different dependent variables (Table 3). There was a significant positive Pearson correlation between pupil accommodation and blink rate (PCCs = 0.814, significance *p* < 0.01). The results indicate that the faster the pupil accommodation, the higher the blink rate under different experimental conditions. In other words, the two objective indexes showed consistency in visual fatigue detection, which means that the objective parameters showed consistency. In contrast, cognitive performance and visual fatigue index were inconsistent (PCCs = 0.359, significance *p* > 0.05). The fact that there is no consistency between the two subjective indicators means that they are not amenable to consistency analysis, which implies that there may not be a relationship between cognitive performance and the visual fatigue index. Objective indicators showed some correlations with cognitive performance and visual fatigue index, respectively. A significant positive Pearson correlation existed between pupil accommodation and cognitive performance (PCCs = 0.398, significance *p* < 0.01). The results showed that under different experimental conditions, the faster the pupil accommodation, the better the cognitive performance. There was a significant positive Pearson correlation between pupil accommodation and visual fatigue index (PCCs = 0.747, significance *p* < 0.01). The results showed that the faster the pupil accommodation, the higher the visual fatigue index under different experimental conditions. Therefore, to address this phenomenon, we took the approach of analyzing the subjective parameters separately.

## 4. Discussion

In this paper, we have examined the impact of text color and ambient illumination on visual fatigue under negative polarity display conditions, with the aim of exploring more ergonomic theory and user preferences for the interface design of visual terminal devices in low-light settings. Data analysis and prior research clearly demonstrate that users experience varying levels of visual fatigue when exposed to different text colors and levels of ambient illumination. This study evaluates and compares both objective factors (eye dynamic change data) and subjective factors (cognitive performance and subjective visual fatigue index) across two dimensions. The following discussion is based on the data analysis results concerning text color, ambient illumination, and their interaction.

### 4.1. Ambient Illumination

Objective indicators of visual fatigue show a decrease as ambient illumination improves, indicating a negative correlation between the two factors—improvements in ambient illumination are associated with reduced visual fatigue. However, this is limited to the changes in our independent variable, ambient illumination, which were not substantial, resulting in insignificant changes in visual fatigue. Nevertheless, it is observable that with increased ambient illumination, visual fatigue improves, particularly in objective parameters, where visual fatigue under 10 lux is less severe than that under 5 lux and 0 lux. These results align with findings from Shieh and Lin [38], Zhang et al. [39], and Wu et al. [40].

In terms of subjective measures, ambient illumination does not exhibit a linear relationship with visual fatigue. For some text colors, cognitive performance, particularly for green text, even declines with increased ambient illumination, while other colors show no significant differences. Regarding the visual fatigue index, there is no significant linear change in perceived visual fatigue with ambient illumination. However, overall data suggest a slightly higher visual fatigue index under 10 lux compared to 0 lux, revealing some inconsistency between subjective and objective parameters under different ambient illumination conditions.

### 4.2. Text Color

In terms of objective factors, the study found that variations in text color significantly influenced visual fatigue, resulting in discernible changes in eye movement data. Notably, red text was associated with a substantial decrease in blink rate and pupil accommodation among subjects, followed sequentially by green, blue, white, and yellow text. Yellow text was found to elicit the highest blink rate and pupil accommodation, indicating that the severity of visual fatigue was not uniform across different colors. Specifically, the levels of visual fatigue experienced by subjects when exposed to various colored texts were ranked as follows: red text > green text > blue text > white text > yellow text. These findings align with those of Tian Peiyuan et al. [41].

Previous research also supports these conclusions; for instance, Osaka proposed that red coloration elicits a greater degree of visual fatigue compared to green and yellow [42]. Similarly, Lin et al. highlighted the significant impact of red color on visual fatigue [18]. However, Nelson et al. reported that while red and green are more likely to induce visual fatigue than other colors, blue appears to cause less fatigue than black [43]. This discrepancy may arise from the specific characteristics of negative polarity text. Although some EEG-based studies have found no significant differences in visual fatigue across various colors [44], both ocular data and subjective assessments indicate that visual fatigue varies markedly among different colors.

Regarding subjective factors, there was no significant variation in cognitive performance among subjects when exposed to yellow, white, and blue text. However, a slight dip in cognitive performance was noted with green text, and a more pronounced decrease was observed with red text. This could be attributed to the “tenseness” of the color red, which may adversely affect cognitive performance [14].

In the subjective visual fatigue index, the degree of visual fatigue of specific text colors, such as red, green, and blue, was subjectively considered by the subjects to produce a higher degree of visual fatigue. This phenomenon may be caused by the subjects being less accustomed to these three text colors. In addition, there is an interesting phenomenon that yellow text was perceived to be the least visually fatiguing in both subjective and objective evaluation criteria, which also happens to be the primary color presented by the eye protection mode involved in hardware companies like Apple Inc. Overall, the objective and subjective metrics show consistency in the effect of text color on visual fatigue.

### 4.3. Interaction between Text Color and Ambient Illumination

From previous studies, ambient illumination with a large gap affects color appearance [45]. Limited by the small variation in ambient illumination we chose, the interaction of ambient illumination and text color did not have a significant effect on visual fatigue, as presented in previous studies.

Among the objective factors, the changes in pupil accommodation and blink rate show significant differences in different-color text with increased ambient illumination. In contrast, in the green text, the change in ambient illumination from 5 lux to 10 lux brought a significant increase in pupil accommodation, while in the other color texts, the change in ambient illumination from 5 lux to 10 lux brought a significant increase. In contrast, no similar phenomenon was observed in the relationship between the other color texts and ambient illumination. In the variation in blink rate, yellow text and red text showed a slightly lower blink rate than 0 lux and 10 lux at 5 lux ambient illumination. Although such a variation was not significant, such a phenomenon was not shown in blue and green, so we speculate that warmer colors may have such a fluctuation in blink rate, while cooler colors may not.

Regarding subjective factors, the interaction effects of ambient illumination and text color on visual fatigue were insignificant, and the effects on cognitive performance were insignificant. They did not show regularity, whereas the subjects’ perception of visual fatigue was related to their evaluation of visual fatigue.

Text color interacted with ambient illumination, and visual fatigue’s subjective and objective parameters appeared inconsistent.

In summary, this paper presents three key findings: First, in negative polarity display mode, variations in text color significantly affect users’ visual fatigue. This difference is evident not only in subjective preferences and self-assessment of visual fatigue but also in objective measures such as pupil accommodation and blink rate. Our comparison with previous studies, which included a broader range of colors, further enriches the understanding of the effect of color. Secondly, under negative polarity display modes, the extent to which ambient illumination changes affect visual fatigue varies significantly depending on the text color. Third, text color under negative polarity has an effect on the user’s cognitive ability.

Based on these findings, we propose several practical applications: Firstly, to reduce visual fatigue for users who must engage with visual device terminals in negative polarity mode over extended periods, altering the text color on interactive interfaces could be beneficial. The design of interfaces for devices like HUDs (Head-Up Displays) could be made more ergonomic by minimizing the use of red and green text colors. Secondly, for professionals who spend considerable time in front of interactive interfaces, adjusting the text color in accordance with the ambient illumination of their workspace could help mitigate visual fatigue and potentially enhance work efficiency, as suggested by studies [46,47]. Thirdly, given that users often utilize visual terminal devices in dark environments, tailored ambient illumination recommendations should be provided to optimize user experience, as indicated by Zhou et al.’s research [48].

The limitations of this study are threefold: Firstly, due to experimental constraints, we were restricted to testing and analyzing data for only five text colors, not the diverse and intricate palette available in interface design. Moreover, the background color for negative polarity text is not only pure black. Secondly, the limited range of ambient illumination levels tested may have restricted our ability to more robustly explore the interactive effects of text color and ambient illumination on visual fatigue; a broader spectrum of illumination levels could have yielded more conclusive results. Thirdly, the objective parameters assessed were limited to eye movement data; the credibility and depth of our findings might have been enhanced by incorporating multi-channel EEG (electroencephalogram) data to measure visual fatigue [49].

## 5. Conclusions

The study’s outcomes indicate a significant impact of negative polarity text color and ambient illumination on visual fatigue under low-illumination conditions, typical of a simulated nighttime environment, with an interactive effect between two factors. With uniform ambient illumination, the severity of visual fatigue follows the order: red text > green text > blue text > white text > yellow text. Specifically, red text is associated with a higher degree of visual fatigue, while yellow text results in the least. Subjective evaluations indicate that users experience more pronounced visual fatigue with red text. In terms of cognitive performance, yellow and white text show significantly better results under ambient illumination that simulates night conditions, outperforming other colors. Enhanced ambient illumination can ameliorate visual fatigue under low-light and negative polarity text conditions; however, the improvement varies among different text colors, leading to diverse self-evaluations of visual fatigue.

This paper addresses the level of visual fatigue experienced by the human eye at night when interacting with visual terminal devices featuring different text colors and ambient illumination. The research presented is application-oriented, particularly in hardware development. Firstly, it is recommended to utilize yellow text or symbols whenever ambient illumination is insufficient at night, minimizing the use of red and green text or symbols. Additionally, designers of visual device terminals could integrate ambient illumination with the display interface, allowing the interface’s color scheme to adapt to the ambient illumination, thereby reducing visual fatigue. Determining a visual fatigue index can also serve to gauge the visual fatigue levels of various visual display terminals in the market, providing valuable information for users seeking devices that minimize visual fatigue, although the questionnaire used for this purpose requires further refinement.

For future research, we aim to refine the following aspects: Firstly, we plan to expand the scope of our color research to include not only static elements like symbols and text but also dynamic content such as animations or videos, to investigate the role of color in inducing visual fatigue across various media. Secondly, we intend to broaden the range of ambient illumination levels studied, moving beyond the constraints of low-ambient-light conditions. Thirdly, we will incorporate EEG-like measuring devices to more accurately track changes in visual fatigue from an objective standpoint and to analyze the cognitive effects of text color using the latest analytical plug-in tools.

## Figures and Tables

**Table 1:** HSB, RGB values of the colors used in the experiment and luminance contrasted with background.

Text Color	HSB	RGB	Luminance Contrasted with Background
White	(0°, 0%, 95%)	(242, 242, 242)	19.60
Yellow	(49°, 70%, 62%)	(158, 138, 47)	6.13
Blue	(218°, 70%, 62%)	(47, 88, 158)	3.01
Green	(113°, 70%, 62%)	(60, 158, 47)	6.12
Red	(3°, 70%, 62%)	(158, 53, 47)	3.00
Background	(0°, 0%, 0%)	(0, 0, 0)	-

**Table 2:** Means and standard deviations of each dependent variable across experimental conditions.

Dependent Variables				
Independent Variables	Pupil Accommodation	Blink Rate	Cognitive Performance	Visual Fatigue Index
White.0 lux	0.0259 (0.0063)	5.343 (0.535)	46.7 (2.16)	5.75 (0.75)
White.5 lux	0.0310 (0.0074)	6.507 (0.535)	47.0 (2.26)	5.40 (1.07)
White.10 lux	0.0310 (0.0075)	6.508 (0.375)	46.2 (2.86)	5.30 (1.13)
Yellow.0 lux	0.0396 (0.0050)	6.709 (0.576)	46.4 (4.74)	5.90 (0.74)
Yellow.5 lux	0.0408 (0.0025)	6.517 (0.558)	47.1 (1.60)	5.75 (0.78)
Yellow.10 lux	0.0489 (0.0052)	6.811 (0.505)	46.4 (4.74)	5.78 (0.74)
Blue.0 lux	0.0144 (0.0045)	4.797 (0.391)	45.2 (2.49)	2.50 (1.11)
Blue.5 lux	0.0190 (0.0075)	4.823 (0.431)	47.1 (1.60)	2.73 (1.22)
Blue.10 lux	0.0200 (0.0066)	5.386 (0.266)	47.0 (2.26)	2.87 (1.16)
Green.0 lux	0.0053 (0.0022)	4.101 (0.670)	45.2 (2.49)	2.52 (0.84)
Green.5 lux	0.0056 (0.0020)	3.987 (0.602)	43.8 (5.83)	2.62 (1.30)
Green.10 lux	0.0097 (0.0033)	4.503 (0.299)	41.6 (4.79)	2.39 (0.87)
Red.0 lux	0.0021 (0.0003)	3.683 (0.310)	42.0 (5.08)	1.58 (0.54)
Red.5 lux	0.0025 (0.0005)	3.834 (0.564)	40.4 (6.04)	1.78 (0.60)
Red.10 lux	0.0030 (0.0006)	3.646 (0.411)	41.6 (4.01)	2.11 (0.64)

**Table 3:** Pearson correlation tests for dependent variables.

	Pupil Accommodation	Blink Rate	Cognitive Performance	Visual Fatigue Index
Pupil Accommodation	1			
Blink Rate	0.814 **	1		
Cognitive Performance	0.398 **	0.403 **	1	
Visual Fatigue Index	0.747 **	0.752 *	0.359	1

* indicates a correlation at the 0.05 level, ** indicates a significant correlation at the 0.01 level.

## Data Availability

Data is contained within the article.
